# Changes in the Number of Medal Events, Sport Events, and Classes During the Paralympic Games: A Historical Overview

**DOI:** 10.3389/fspor.2021.762206

**Published:** 2022-01-20

**Authors:** Julia Kathrin Baumgart, Eline Renee Blaauw, Roy Mulder, Anna Cecilia Severin

**Affiliations:** Centre for Elite Sports Research, Department of Neuromedicine and Movement Science, Norwegian University of Science and Technology, Trondheim, Norway

**Keywords:** adaptive sports, disability, IPC, Para sports, categorization

## Abstract

**Purpose:**

To chart how changes in the number of medal events relate to changes in the number of sport events and classes during the Paralympic Games (PG) between 1960 and 2018.

**Methods:**

Web-scraping was used to extract information from the website of the International Paralympic Committee (IPC) on all unique medal events, sport events, and classes per PG, which were then accumulated per sport to descriptively identify and further explore changes.

**Results:**

The increased number of medal events during the early Summer Games (SG) (1960–1984: 113–975) and Winter Games (WG) (1976–1994: 55–113) was primarily due to an increased number of classes and sport events. While this suggested an increased sports participation among athletes with disabilities, it made the PG difficult to organize. A decrease in the number of medal events subsequently occurred during the SG (1984–1992: 975–489) and WG (1994–2006: 133–58). This was mainly achieved by reducing the number of sport events in the larger sports. Following this decline phase, the number of medal events and sport events has remained relatively stable for both editions of the PG, though this was achieved through different strategies. The WG employed the time-factor system for all individual sports, which enabled competitions across classes within sport events and thus, award a single gold medal (one medal event) for several classes. The SG have maintained the number of medal events despite a slight increase in classes (112–181). This was due to some sports combining classes in the same event, while others excluded certain classes from certain sport events.

**Conclusions:**

The number of medal events during each PG appear to be closely related to the number of sport events and, partially, to the number of classes. The stability in the number of medal events may indicate that a balance has been achieved, where there currently are enough classes and sport events to ensure fairness, while also maintaining a level of prestigiousness for winning a medal. However, it remains to be seen whether this stability will last or if the continued growth of the PG with more athletes and countries will warrant changes in the number of medal events.

## Introduction

The Paralympic Games (PG) are organized every 2 years, alternating between the Summer (SG) and Winter Games (WG), where athletes with certain physical and intellectual disabilities compete across multiple sports. The PG's origin is the Stoke Mandeville Games, which was first organized in 1948—at the same time as the Olympic Games—and featured 16 athletes with spinal cord injury competing in archery. The Stoke Mandeville Games grew in the number of participating athletes and countries and later became the PG. The first international SG were held in 1960 in Rome, with 209 competitors across seven sports (www.paralympic.org/rome-1960/results). Sport participation for people with a disability was at that time predominantly used for rehabilitation, but participation has been steadily increasing and athletes who qualify for the PG are now considered elite level (McCann, 1996; Gold and Gold, 2007; Vanlandewijck and Thompson, 2011). The PG are one of the largest sporting events in the world; at the 2016 SG in Rio de Janeiro, there were 4,327 competitors across 22 sports, and at the 2018 WG in PyeongChang there were 566 competitors across six sports (www.paralympic.org/results).

The gateway to participation in the PG has always been through a classification process, where athletes are grouped together for competition based on observable, disability-related properties that they have in common (Tweedy and Vanlandewijck, 2011). Sherrill (1999) stated that classification aims to “*ensure that winning or losing an event depends on talent, training, skill, fitness, and motivation rather than unevenness among competitors on disability-related variables*.” Athletes were originally classified based on their medical diagnosis, with a gradual shift being initiated in 1992 toward today's *sport-specific functional* classification system, where athletes are classified based on the functional impact their disability has on performance in a specific sport (Legg and Steadward, 2013). However, it is challenging to accurately establish the degree to which disability-related properties affect performance during competition, and the classification system therefore continues to evolve (Howe and Jones, 2006).

Like the athletes competing in the Olympic Games, Paralympic athletes participate in several sport events, where the winners are identified through a ‘final race’ or a medal event. The International Olympic Committee (IOC) defines a sport event as “a competition in a sport that gives rise to a ranking” (https://www.olympic.org/faq/sports-programme-and-results/). Unlike the Olympic Games, which only award two gold medals per sport event (men and women), the PG often award one gold medal per class in each sport event. It is therefore not surprising that both changes to how the classes are set up and the number of sport events in each sport have a direct impact on the number of medal events.

The International Paralympic Committee (IPC) was established in 1989 and has been central to evolving the classification system across sports (Tweedy and Vanlandewijck, 2011; Gérard and Zintz, 2017), while simultaneously engaging in efforts to increase professionalization, media attention and commercialization of sport for athletes with a disability (Gold and Gold, 2007; Pappous et al., 2011; IPC, 2019; Flindall, 2020). During the first editions of the SG and WG, an increased number of sports, sport events, and classes was needed to ensure fairness amongst an increasing number of competitors with different disabilities (Gold and Gold, 2007). However, this resulted in a high number of medal events which created organizational challenges and hampered both media coverage and spectator experience (Wu, 1999; Howe, 2008; IPC, 2009). Efforts were therefore made to reduce the number of medal events. Even though it was speculated that this was achieved through reductions in the number of classes (Mauerberg-Decastro et al., 2016), how changes in the number of classes and sport events contributed to this is not entirely clear.

A close collaboration between the IPC and the IOC was formalized in 2001 and has been essential in further promoting the high-level performances of athletes with disabilities. This was achieved through increased financial support and media coverage, as well as access to using the same venues, infrastructure, and levels of planning as the Olympic Games (Bailey, 2008; IPC, 2012). At the same time, it has been argued that the IPC's autonomy has been compromised due to the organization and media coverage of the Olympic Games and PG as one entity (Purdue and Howe, 2012). However, the extent to which organizational changes have influenced the number of medal events, sport events, and classes has not yet been investigated.

This overview aimed to chart how changes to the number of sport events and classes have affected the number of medal events during the PG between 1960 and 2018. An additional analysis was performed to investigate whether changes in the proportion of participating male and female athletes, and the number of competing countries influenced the number of medal events in the same time period.

## Methods

### Data Extraction

The IPC website contains information about all PG in terms of listing the participating sports, countries, and athletes, and detailing the number and names of the medal events (www.paralympic.org/results). The definitions used in this study for the terms “medal event,” “sport event,” and “class” are presented in Table 1.

**Table 1:** Definitions of the terms “medal event”, “sport event,” and “class.”

	**Definition**	**Example from Para swimming**	**Example from Para alpine skiing**
Sport event	An event within a sport	e.g., 50 m backstroke or 100 m freestyle	e.g., giant slalom or downhill
Class	A group of athletes with similar observable, disability-related properties	e.g., S1–S10 for physically impaired and S11–S13 for visually impaired athletes	e.g., LW1–9 for physically impaired standing and LW10–12 for physically impaired sitting athletes
Medal event	Final race or game to determine the medalling athletes/teams of each class within a sport event	e.g., 50 m backstroke S1	e.g., giant slalom LW1

Scripts for web-scraping online information were written in Phython based on the *BeautifulSoup* library (Python Software Foundation, Beverton, OR, USA) and in R (R Core Team, 2013) based on the *rvest* and *dplyr* packages. These scripts were used to extract information of all medal events (Python script), as well as participating athletes (including information on their sex) and countries (R script) for each sport during each PG between 1960 and 2018.

### Data Processing

Further processing of the extracted data was performed in Microsoft Excel Version 1908 (Microsoft Corp., Redmond, WA, USA) and R. For each PG, based on the names of the unique medal events, the unique sport events and classes were manually identified for each sport in Microsoft Excel. In sports where different classes competed together in one sport event, all participating classes were considered when identifying the total number of classes for that sport. Furthermore, the number of unique medal events per sport and PG, was split into female-athletes-only, male-athletes-only, or mixed competitions in R. In addition, for each sport and PG, the number of participating athletes and their sex, as well as countries were identified in R. Whenever the data extracted from the IPC's website was incomplete or needed verification, additional input was provided by records (e.g., rules and regulations, operational and classification manuals of the sport in question). The data processing of the information extracted from the website was approved by the Norwegian Centre for Research Data (ID: 967492).

The numbers presented in the figures provide a descriptive overview over how the largest summer and winter sports contribute to the changes in the medal events, sport events, and classes. To show the impacts of largest sports clearly, all appearances by athletes and countries across multiple sports were counted (i.e., a country with athletes in three different sports at the same PG, was counted three times). The same method was used in terms of showing the effect of sex, where athletes were counted more than once if they participated in more than one sport at the same PG. This was done to be able to indicate the medalling chances for female and male competitors.

## Results

**Figure 1:**
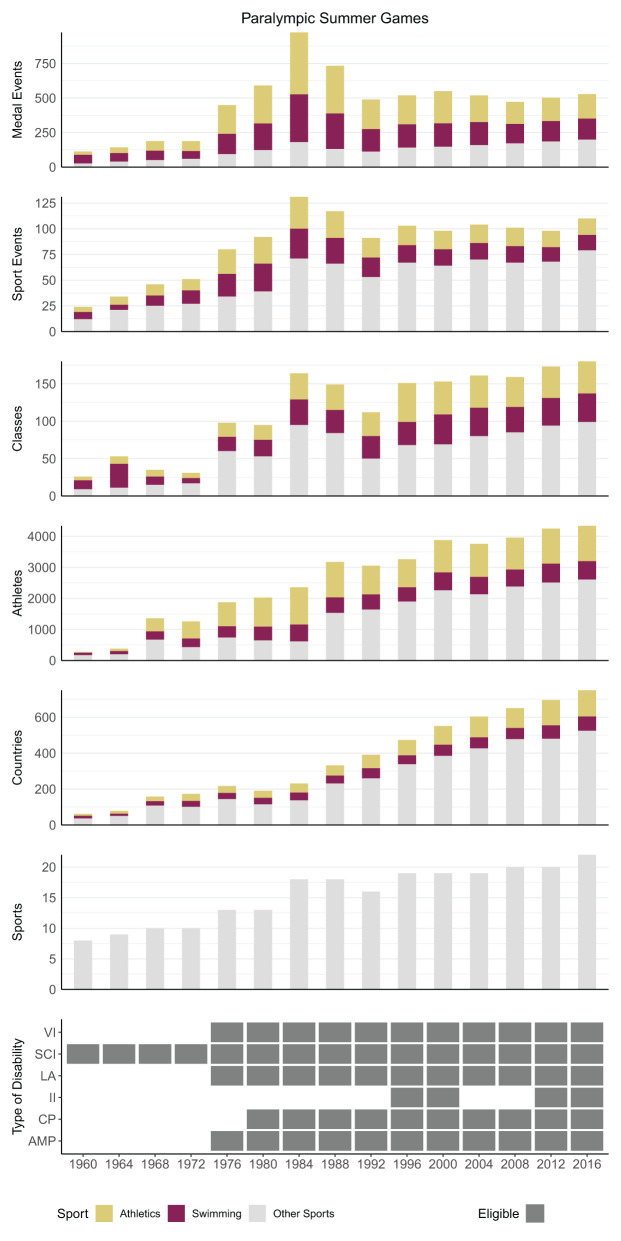
Schematic overview showing the contribution of swimming and athletics to the total number of medal events, sport events, and classes during each of the Paralympic Summer Games. Some sports have the same division of classes; in these cases, the number of classes is counted once per sport. In addition, some athletes and countries participated in more than one sport; in these cases, the number of athletes and countries were counted once per sport. VI, visual impairment; SCI, spinal cord injury; other impairments (LA, les autres); II, intellectual impairment; CP, cerebral palsy; AMP, amputation. For the number of unique countries, (see Table 2).

**Figure 2:**
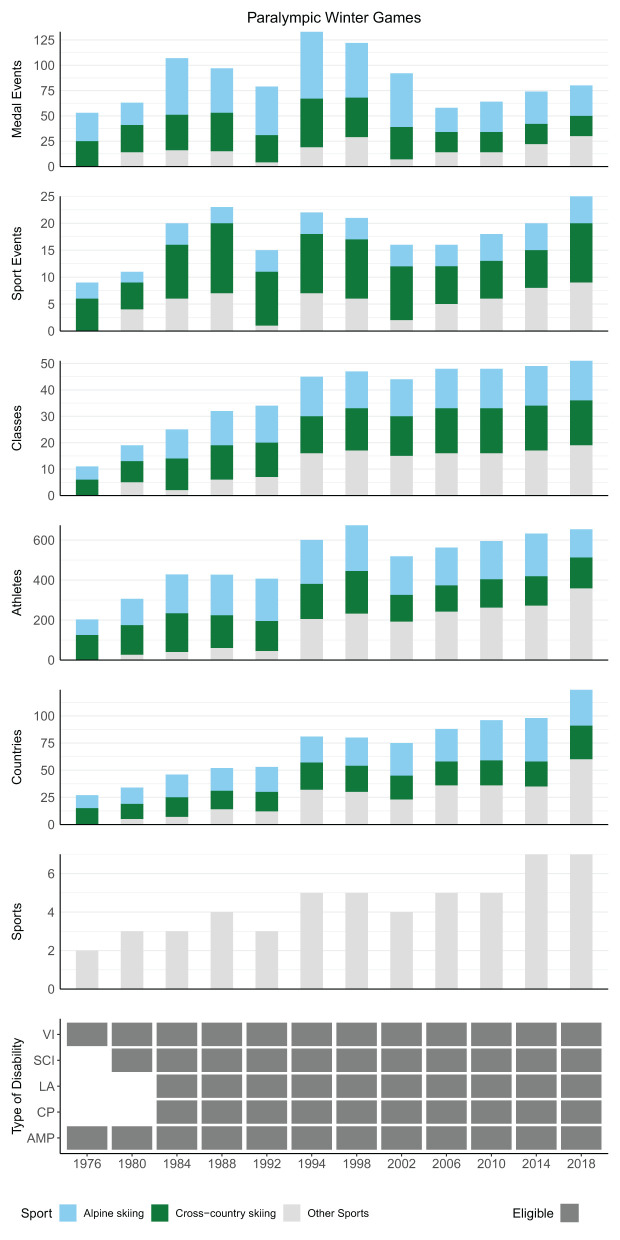
Schematic overview showing the contribution of cross-country skiing and alpine skiing to the total number of medal events, sport events, and classes during each of the Paralympic Winter Games. Some sports have the same division of classes; in these cases, the number of classes was counted once per sport. In addition, some athletes and countries participated in more than one sport; in these cases, the number of athletes and countries were counted once per sport. VI, visual impairment; SCI, spinal cord injury; other impairments (LA, les autres); CP, cerebral palsy; AMP, amputation. For the number of unique countries, (see Table 3).

The number of medal events, sport events, and classes were obtained for each of the 28 summer sports and seven winter sports that have ever been included in the PG. Most of these sports are currently included; 23 summer sports were represented at the 2016 Rio Games, and six winter sports at the 2018 PyeongChang Games. When looking at how the number of medal events during the SG have changed over the years, there is an incline from 1960 to 1984, followed by a decline from 1984 to 1992 (Figure 1). For the WG, the number of medal events appears to fluctuate more, but, similar to the SG, an incline can be seen from 1976 to 1994, followed by a decline from 1994 to 2006 (Figure 2). Over the more recent years (since 1992 for SG, and 2006 for WG), the number of medal events have remained fairly stable, with only minor fluctuations. Both editions of the PG have two sports that are dominating the number of medal events, sports events, and classes—swimming and athletics for the SG (Figure 1), and alpine and cross-country skiing for the WG (Figure 2).

**Table 2:** Overview of the number of unique countries, and number of countries counted once per sport for each edition of the Paralympic Summer Games.

**Year**	**Location**	Number of unique countries^*^	**Total number of country entries**
1960	Rome	17	61
1964	Tokyo	20	79
1968	Tel Aviv	28	158
1972	Heidelberg	41	173
1976	Toronto	41	217
1980	Arnhem	42	191
1984	Stoke-Mandeville	54	231
1988	Seoul	60	332
1992	Barcelona-Madrid	84	391
1996	Atlanta	104	473
2000	Sydney	123	551
2004	Athens	135	604
2008	Beijing	146	651
2012	London	164	696
2016	Rio de Janeiro	160	750

* *The numbers of the unique countries are based on the overview numbers provided per Game by the IPC (e.g., www.paralympic.org/rome-1960/results)*.

**Table 3:** Overview of the number of unique countries, and number of countries counted once per sport for each edition of the Paralympic Winter Games.

**Year**	**Location**	Number of unique countries^*^	**Total number of country entries**
1976	Örnsköldsvik	16	27
1980	Geilo	18	34
1984	Innsbruck	21	46
1988	Innsbruck	22	52
1992	Tignes-Albertville	24	53
1994	Lillehammer	31	81
1998	Nagano	31	80
2002	Salt Lake City	36	75
2006	Turin	38	88
2010	Vancouver	44	96
2014	Sochi	46	98
2018	PyeongChang	49	124

* *The numbers of the unique countries are based on the overview numbers provided per Game by the IPC (e.g., www.paralympic.org/ornskoldsvik-1976/results)*.

An overview of the number of medal events, sport events and classes, participating athletes, and countries in each sport during each PG is available as Supplementary Material 1 (Excel file).

### Summer Games

Between 1960 and 1984 (*incline phase*) the number of medal events increased eight-times (113–975). During the incline phase, both the number of sport events (24–129) and classes (26–164) increased considerably (Figure 1). During this time, swimming and athletics together contributed to 82% of the increases in medal events, 45% of the sport events, and 38% of the classes.

Between 1984 and 1992 (*decline phase*), the total number of medal events decreased (from 975 to 489), which coincided with fewer sport events (129–91) and classes (from 164 to 112). During this time, athletics and swimming together accounted for 86% of the decrease in the total number of medal events, 55% of the decrease in sport events, and 14% of the decrease in classes. This shows that the reduced number of medal events was mainly facilitated by a reduction in sport events and less so in classes in these larger sports. Most sports also regulated the number of medal events by limiting participation in certain sport events to some of the classes.

Between 1992 and 2016, the number of medal events (range of absolute Δyear-to-year: 25–47) and sports events (range of absolute Δyear-to-year: 3–12) remained relatively stable (*stable phase*), despite a steady increase in the number of classes (112–181). The increased number of classes was caused mainly by the inclusion of seven new sports (i.e., basketball ID, canoeing, football 5-a-side, rowing, sailing, triathlon, and wheelchair rugby), which added 35 classes. However, the increased number of classes was not reflected in the number of medal events, since classes started to compete together in certain sport events, and not all classes participated in all sport events (and thus, medal events).

**Figure 3:**
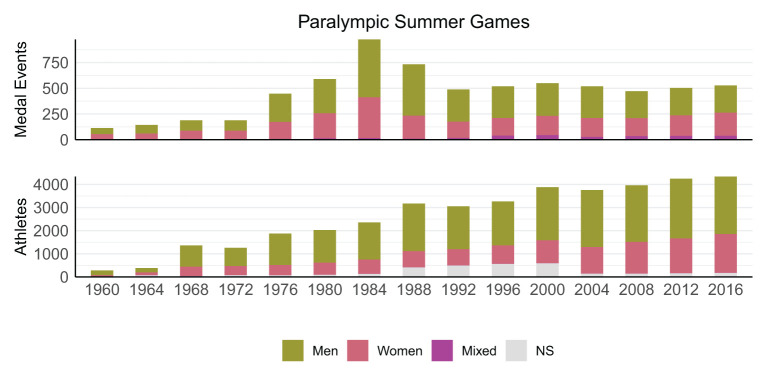
Schematic overview showing the split between medal events for female-only, male-only, and mixed competitions, as well as the number of female and male athletes during each of the SG. Some athletes participated in more than one sport; in these cases, the number of athletes were counted once per sport.

Irrespective of the three-phase pattern in the medal events, the number of participating athletes and countries in the SG has steadily increased from 1960 to 2016. The opportunity to win a medal was disproportionally higher for athletes participating in swimming or athletics (39–74% of the athletes competing in swimming or athletics won 62–82% of the medals). Furthermore, the number of medal events for female athletes was disproportionally larger than the number of female athletes (22–39% of the competing athletes were female and won 31–47% of the medals) (Figure 3).

### Winter Games

Between 1976 to 1994 (*incline phase*), the number of medal events increased 2.5-times (from 53 to 133), which coincided with a slight increase in the number of sport events (from 9 to 22) and an increase in the number of classes (from 11 to 45) (Figure 2). Biathlon was introduced in 1988, which increased both the number of classes (+10) and sport events (+1) and subsequently also medal events (+13). Further, cross-country skiing introduced short- and middle-distance races, which also increased the number of sport events (+5). There was a small decrease in medal events between 1988 and 1992 (from 97 to 79), followed by an increase between 1992 and 1994 (from 79 to 133). This can almost solely be attributed to the absence of ice sledge speed skating at the 1992 WG. This shows that the number of medal events can be heavily affected by the inclusion or exclusion of a single sport.

Between 1994 and 2006 (*decline phase*), the number of medal events decreased (from 133 to 58), with only slight reductions in the number of sport events (from 22 to 16), while the number of classes remained relatively stable (range: 45–48) (Figure 2). The reduction in sport events was because ice sledge speed skating was permanently removed in 2002. Further, all three remaining individual sports (i.e., biathlon, cross-country skiing, and alpine skiing) employed a factor-based system in 2006. Under this system, which remains active today, all athletes compete within a category (e.g., three categories in cross-country skiing for physically impaired standing, physically impaired sitting, and visually impaired skiers). While athletes in these categories are further divided into classes, each athlete is assigned a class-specific time-factor which allows all athletes within the category to compete for one medal. There is thus a single medal event per category (instead of class) for each sport event.

While the number of medal events have been relatively stable since 2006, it will be interesting to see how the WG develop over the coming years. For the period between 2006 and 2018, there was a slight increase in medal events (from 58 to 80) and sport events (from 16 to 25), mainly due to the inclusion of snowboard and wheelchair curling. The number of classes in the WG (range: 48–51) remained relatively stable.

**Figure 4:**
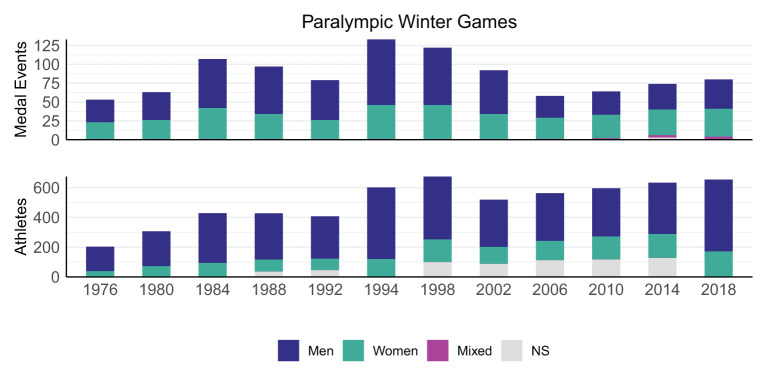
Schematic overview showing the split between medal events for female-only, male-only, and mixed competitions, as well as the number of female and male athletes during each of the Paralympic Winter Games. Some athletes participated in more than one sport; in these cases, the number of athletes were counted once per sport.

Although not as clear as for the SG, the number of participating athletes and countries in the WG has generally also typically increased since 1976. Similar to the SG, the number of medal events for cross-country and alpine skiing was disproportionally larger than the number of participating athletes in these two sports (45–100% of the athletes competing in cross-country or alpine skiing won 63–100% of the medals). Furthermore, the share of medal events for female athletes was also disproportionally larger than the share of participating female athletes for the WG (19–26% of the competing athletes were female and won 33–50% of the medals) (Figure 4).

## Discussion

This study provides a historical overview of how changes to the number of sport events, and classes at the PG has related to changes in the number of medal events. There was initially an increase in the number of sport events and classes, which resulted in an increased number of medal events, and caused challenges in terms of organization, media coverage, and public interest. Accordingly, the number of medal events were gradually decreased over the next editions, mainly through fewer sport events in the larger sports. Since then, both the number of medal events and sport events have remained stable with only minor changes. In the following, we discuss some of the reasons that led to changes in the classes and sport events, and thereby medal events.

For both the SG and WG, there were two primary drivers for the initial increase in medal events (*incline phase*): (1) The eligibility of more athletes with a wider range of disabilities, which subsequently increased the number of classes; and (2) the inclusion of new sports and more sport events in existing sports. In brief, the first four PG were organized in the summer and only allowed athletes with a spinal cord injury to compete (Gold and Gold, 2007; Tweedy and Vanlandewijck, 2011). The first WG was held in 1976 and only allowed athletes with an amputation or visual impairment to compete. Athletes with other types of disabilities (i.e., cerebral palsy, intellectual impairment, and other disabilities/*les autres*) were gradually allowed to participate in both editions of the PG between 1976 and 1984 and sub-classes were added under each medical diagnosis to ensure fair competitions for the increasing number of athletes with different disabilities (Wu, 1999; Gold and Gold, 2007). A more in-depth description of the development of and changes to the organizations advocating for people with different disabilities can be found elsewhere (Tweedy and Vanlandewijck, 2011; Gérard and Zintz, 2017).

The increased number of sport events during both editions of the PG was in part due to the inclusion of new sports, to cater for the needs of athletes with larger variety of disabilities. Examples of this are goalball for athletes with a visual impairment, boccia for athletes with severe cerebral palsy and basketball ID for athletes with intellectual impairments. In addition, new sports were also added to better align the Olympic Games and PG, such as rowing, sailing, triathlon, and snowboard. Furthermore, existing sports such as athletics added sport events (e.g., long jump, high jump, and triple jump). Ultimately, the growth of the PG through the inclusion of more athletes with a wider range of disability types was reflected in the increasing number of medal events (Mauerberg-Decastro et al., 2016). However, it was recognized that the prestigiousness of winning a medal started to deteriorate since few athletes (occasionally, a single athlete) competed within each class and medal event (Wu, 1999). Further, the administration processes of organizing so many competitions were inefficient and complicated (Wu, 1999; Howe and Jones, 2006), which often resulted in competitions being canceled. In addition, the large number of medal events made it difficult for the media to cover all competitions, and thus, for the spectators to follow the PG (Wu, 1999). These are likely the primary reasons why it was decided to reduce the number of medal events in the years to come.

While the decrease in medal events corresponded with a relatively large decrease in sport events in the larger sports during the SG (from 1984 to 1992), there was only a small corresponding reduction for the WG (from 1994 to 2006). Surprisingly, both editions of the PG saw the number of classes remain relatively stable, especially in the larger sports. Much of the initial decrease in medal events during the SG was caused by the cancellation of 150 medal events in Seoul in 1988. This has been anecdotally linked to insufficient numbers of athletes in some classes and sport events, due issues in the classification process^1^. Thereafter, it was mainly a rather systematic drop in sports events in swimming and athletics that caused the reducing number of medal events. For example, swimming excluded all 25-m distances to align the competition with the Olympic Games and to comply with the 50-m pool standard for international competitions, which also facilitated competition at the same venues. Similarly, athletics excluded the short sprint distances (i.e., 20, 60, and 80 m), and reduced the number of jumping events and throwing events. During the WG, the small reduction in sport events from the 2002 Games was because ice sledge speed skating was permanently removed to align with the Olympic Games (Vanlandewijck and Thompson, 2016).

It was also during the decline phase that a push was initiated by the IPC toward introducing *sport-specific functional classification systems*. Since this time classification was increasingly based on the functional capabilities needed to perform a given sport rather than the type of disability, thus allowing athletes with different disabilities to compete against each other. Swimming, alpine skiing, and cross-country skiing were among the first sports to make this transition. However, the expected reductions in the number of classes (and consequently medal events) did not occur in these sports. In addition, swimming and athletics had several interest groups that were active under the IPC during the decline period (Gérard and Zintz, 2017), which may have given these sports a certain leverage for maintaining a larger share of the medal events compared to the smaller sports. Accordingly, the number of medal events per participating athlete is now disproportionally larger in swimming and athletics. This may hamper the development of medalling chances in other sports since countries are often inclined to support their national sports federation based on success at larger international competitions.

During the SG, in addition to the reduction in sport events, sports began to restrict which classes could compete in certain sport events. The WG took a different approach and employed a factor-based system in the individual sports (Rosso and Gastaldi, 2020), which allowed them to maintain the number of classes while still decreasing the number of medal events. The factor-based system simplifies the organizational procedures, media coverage, and spectator appeal of these sports. It may, therefore, be preferable to a system with more classes competing separately, especially in smaller sports, where there are relatively few athletes competing in each class. However, the time-factor system has disadvantages, primarily since it is not necessarily the athlete who crosses the finish-line first who wins and finalizing the results can take a considerable amount of time. This leaves both athletes and spectators unaware of the position of athletes during the race and the final rank, which may have a negative effect on the appeal of the sport. It appears that the SG and WG employed different strategies to decrease the number of medal events during this period, without primarily altering the number of included classes.

The PG have entered a phase where the number of medals events remains relatively stable (SG since 1992 and WG since 2006). This may indicate that they have, at last, achieved a good balance where there are enough classes and sport events to ensure fairness, while still maintaining a level of prestigiousness for winning a medal. Notably, the 2016 Summer edition saw the highest number of classes since the start of the PG, which indicates that more classes were needed in some sports to maintain fair competitions for the increasing number of participating athletes. To keep the number of medal events as low as possible, classes were excluded from participation in certain sport events. Which classes were allowed to compete in which sport events seems rather arbitrary and may have led to athletes not being allowed to compete in their main event. With continued growth in the PG (particular the SG), and rising numbers of participating athletes and countries, this strategy may become challenging to maintain. Even with the current size, there are challenges with organizing the numerous medal events and securing sufficient media attention and an easy-to-follow spectator experience (IPC, 2011, 2019), and these may not get any less.

Interestingly, both the SG and WG have managed to keep the number of medal events relatively stable over the recent years, despite the continued increase in the number of participating athletes and nations. A consequence of this is increased competition for and prestigiousness of winning a medal, which aligns with the IPC's and IOC's focus on promoting high-level performance. At the same time, the IPC has been working toward developing opportunities of female athletes and athletes with high support needs in sport at all levels and in all structures. While the numbers provided in the current study indeed support that the IPC has succeeded in providing sufficient medal opportunities for female competitors, it remains unclear if this is the case for athletes with more severe impairments. In fact, it has been argued that medal opportunities and media coverage of athletes with severe impairments are insufficient, which has been related to their perceived lack of “aesthetically pleasing sporting performances” by broadcasters and the general spectator (Purdue and Howe, 2012, 2013).

### Limitations and Future Directions

It must be acknowledged that the reasons behind the changes in the numbers of medal events, sport events, and classes are complex and multi-faceted. The growth in the popularity of the PG must be considered, along with corresponding increases in the number and professionalism of participating athletes, global finances, and (social) media coverage, while at the same time needing to balance a push toward fewer medal events with maintaining fairness amongst competitors. While this was beyond the scope of the current overview, more in-depth investigations of these factors should be addressed in future research.

Furthermore, the data used in this overview is based on the official records provided on the IPC website, which may not necessarily be complete, especially during the earlier PG. Data from the earlier editions of the PG has been transferred to online databases retrospectively and may therefore be subject to errors. Therefore, whilst efforts were made to ensure the quality of the included data, it is possible that minor inaccuracies may have been included in this overview. In this context, due to lack of validated information, the number of classes for volleyball are only provided and included for 2012 and 2016. While this did not allow us to show an entirely complete picture, we assume that the number of classes ranged from 2 to 5, and that not including these numbers did not affect the overall picture.

## Conclusion

The increasing number of medal events during the early SG (until 1984) and WG (until 1994) was due to more classes and sport events being included. While this encouraged sports participation for athletes with disabilities, it made the PG difficult to organize. A decrease in the number of medal events subsequently occurred between the 1984 and 1992 SG and the 1994 and 2006 WG. This was mainly achieved by reducing the number of sport events in the larger sports and, to a lesser extent, by reducing the number of classes in the smaller sports. Following this decline phase, the number of medal events and sport events has remained relatively stable for both editions of the PG, though this has been achieved through different strategies. The WG employed the time-factor system for all individual sports, which has enabled athletes to compete across classes within a sport event and thus, awards a single gold medal (one medal event) for several classes. The SG has seen a slight increase in the number of classes, which has not been reflected in an increased number of medal events. This was either because classes were combined in the same sport event, or that some classes did not compete in certain sport events. The stability in the medal events may indicate that the PG have, at last, developed a balanced system where there are enough classes and sport events to ensure fairness, while still maintaining a level of prestigiousness for winning a medal. Yet, with expectations of continuously increased popularity of the PG and rises in the number of participating athletes and countries, it remains to be seen whether the current strategies to control the number of medal events will suffice or require revision in the future.

## Data Availability Statement

All data that was used in the current study is available online on the IPC's website (http://www.paralympic.org/results). The data processing of the information extracted from the website was approved by the Norwegian Centre for Research Data (ID: 967492).

## Author Contributions

Conceptualization, methodology, formal analysis, and investigation: ERB, ACS, RM, and JKB. Writing—original draft preparation: ERB and JKB. Writing—review and editing: ACS and RM. All authors contributed to the article and approved the submitted version.

## Conflict of Interest

The authors declare that the research was conducted in the absence of any commercial or financial relationships that could be construed as a potential conflict of interest.

## Publisher's Note

All claims expressed in this article are solely those of the authors and do not necessarily represent those of their affiliated organizations, or those of the publisher, the editors and the reviewers. Any product that may be evaluated in this article, or claim that may be made by its manufacturer, is not guaranteed or endorsed by the publisher.

## Footnotes

^1^http://www.chinadaily.com.cn/paralympics/2008-08/29/content_6981819.htm; https://www.paralympicheritage.org.uk/seoul-1988-paralympic-summer-games.

## Supplementary Material

In addition, an online repository was created to provide a more detailed overview including the names of the sport events and classes in each sport, and is available at https://datastudio.google.com/s/nduGA2-GGbM.
